# Application of osteoinductive calcium phosphate ceramics in giant cell tumor of the sacrum: report of six cases

**DOI:** 10.1093/rb/rbac017

**Published:** 2022-04-11

**Authors:** Yitian Wang, Xiangfeng Li, Yi Luo, Li Zhang, Hezhong Chen, Li Min, Qing Chang, Yong Zhou, Chongqi Tu, Xiangdong Zhu, Xingdong Zhang

**Affiliations:** 1 Department of Orthopedics, Orthopedics Research Institute, West China Hospital, Sichuan University, Chengdu 610041, China; 2 Bone and Joint 3D-Printing & Biomechanical Laboratory, Department of Orthopedics, West China Hospital, Sichuan University, No. 37 Guoxuexiang, Chengdu 610041, Sichuan, China; 3 National Engineering Research Center for Biomaterials, Sichuan University, Chengdu 610064, China; 4 Sichuan Baiameng Bioactive Materials Limited Liability Company, No. 37 Guoxuexiang, Chengdu 610041, Sichuan, China

**Keywords:** calcium phosphate, bioceramics, giant cell tumor, sacrum, osteoinductivity

## Abstract

This study aimed at evaluating the possibility and effectiveness of osteoinductive bioceramics to fill the tumor cavity following the curettage of sacral giant cell tumor (GCT). Six patients (four females and two males, 25–45 years old) underwent nerve-sparing surgery, in which the tumor was treated by denosumab, preoperative arterial embolization and extensive curettage. The remaining cavity was filled with commercial osteoinductive calcium phosphate (CaP) bioceramics, whose excellent osteoinductivity was confirmed by intramuscular implantation in beagle canine. All patients were followed by computed tomography (CT) scans postoperatively. According to the modified Neer criterion, five cases obtained Type I healing status, and one case had Type II. At the latest follow-up, no graft-related complications and local recurrence were found. The CT scan indicated a median time of healing initiation of 3 months postoperatively, and the median time for relatively complete healing was 12 months. The excellent bone regenerative ability of the ceramics was also confirmed by increased CT attenuation value, blurred boundary and cortical rim rebuilding. In conclusion, osteoinductive CaP bioceramics could be an ideal biomaterial to treat the large remaining cavity following extensive curettage of sacral GCT. However, further investigation with more cases and longer follow-up was required to confirm the final clinical effect.

**Figure:**
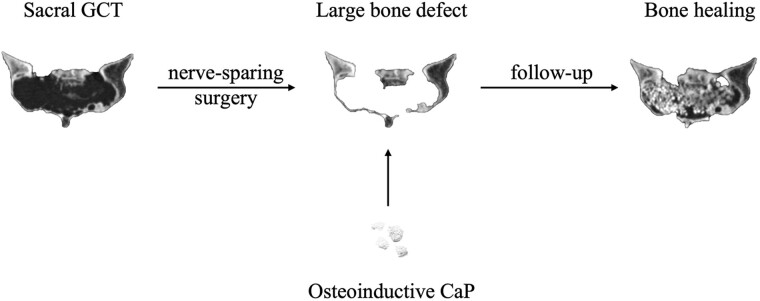

## Introduction

Surgical management of sacral giant cell tumor (GCT) is difficult due to neurologic involvement, extensive bone destruction, complex anatomical structures, and intraoperative blood loss. The management goal of sacral GCT is still challenging and cannot be fully achieved as long bones [1, 2]. The overall complication rate is higher after marginal or *enbloc* resection and subsequent reconstruction compared to curettage, which includes neurologic damage, infection and aseptic loosening [2]. With the advance in surgical technology and denosumab treatment, nerve-sparing surgery has been adapted to sacral GCT to salvage nerve roots and prevent pelvic instability [3, 4].

The bone defect following nerve-sparing surgery is characterized by irregular shape, large volume and surrounding nerve roots [5, 6]. Therefore, it is controversial how to manage the remaining cavity after extensive curettage. There are types of commonly used bone grafts including polymethylmethacrylate (PMMA) [7], autograft [8], allograft [9] and other artificial bone substitutes [10–12]. Because of direct weight-bearing and early radiographic detection of recurrence, PMMA was often used to fill the remaining cavity in the limb [13, 14]. However, in GCT of sacrum, thermal damage could limit its application as it is close to nerve roots. Additionally, secondary osteoarthritis was detected with PMMA packing following curettage [15]. Harvesting autograft is particularly associated with a limited amount of bone and donor site complications [16]. Also, the use of allograft is limited by immunological reaction rejection and fracture [14, 17, 18]. Other bone substitutes also have unique advantages but also inevitable disadvantages [10–12]. Therefore, the gold standard for remaining cavity management in sacral GCT has not yet been established [19].

Biomaterial is defined as a non-drug substance suitable for inclusion in systems that augment or replace the function of body tissues or organs [20]. Great advancement in biomaterial study has promoted the development of regenerative medicine, making it possible to achieve the regenerative repair of bone defects. Thus far, the knowledge on biomaterial has evolved from initial bioinert to subsequent bioactivity, and now biofunctionality, i.e. inducing bone regeneration. As one of the most promising functional biomaterials, calcium phosphate (CaP) ceramics, including hydroxyapatite (HA), β-tricalcium phosphate (β-TCP) and their compounds, have been widely investigated and successfully used for clinical application because of similar inorganic components with natural bone. CaP ceramics have been found with excellent biocompatibility, osteoconductivity and osteoinductivity, which is influenced by many material factors [21, 22]. Generally, the properly interconnected porous structure and surface micro- and nano-topography provide CaP ceramics with superior osteoinductivity [23]. The highly porous scaffolds allow mass transport, cell adhesion and bone ingrowth, without adding any exogenous cell and growth factors [20]. In practice, its effcacy in guiding bone regeneration was confirmed not only by the segmental bone defect in animal models but also by the calvarial defects in children [22, 24]. Therefore, CaP ceramics may have the potential to enhance bone formation on the sacrum following nerve-sparing surgery. To test this assumption, a commercially available osteoinductive CaP bioceramics was used in this study. Its filling effect was remarkable in the short-term follow-up.

## Materials and methods

### Materials

The commercial CaP ceramics (Trade name: osteoinductive CaP bioceramics) were provided by Sichuan Baiameng Bioactive Materials LLC (Original name: Engineering Research Center in Biomaterials, Sichuan University, Chengdu, China). The ceramic was composed of HA and β-TCP (in ∼60%:40% ratio), and the allowable content of other impurities is <5.0%. The phase composition, surface morphology and porosity were analyzed by X-ray diffraction (XRD), field emission scanning microscopy (S4800, Hitachi, Japan) and mercury porosimetry (AutoPore IV 9500, Micromeritics), respectively.

### Animal experimental study

The animal experimental procedure was approved by the Animal Care and Use Committee of Sichuan University, China, following the Guide for the Care and Use of Laboratory Animals published by the Chinese National Academy of Sciences. Six healthy beagles (8–8.5 kg) were used in this study. Intramuscular implantation was performed to evaluate the osteoinductivity of the CaP ceramic sample. Briefly, each beagle was anesthetized with 3% pentobarbital sodium in the prone position with a dose of 30 mg/kg. Then, three linear incisions (2 cm) were made on the bilateral spine (*n* = 3). The dorsal muscle on each side was filled with porous scaffolds (one per incision). The deep fascia was sutured closely. Related complications were assessed every week postoperatively. After 3 months, all samples were harvested and stored in 4% phosphate-buffered paraformaldehyde solution for 7 days before further evaluation.

### Clinical study

In total, six patients, who underwent nerve-sparing surgery of sacral GCT and osteoinductive CaP ceramic filling between 10 January 2019 and 20 April 2020, were retrospectively reviewed. The inclusion criteria included: (i) the histologically proven sacral GCT with sacral nerves involvement (S1–S3), (ii) underwent nerve-sparing surgery and (iii) no previous surgical treatment. The exclusion criteria included: (i) multicentric GCT and sarcomatous change and (ii) lack of regular follow-up. This retrospective study was performed according to the principles embodied in the Declaration of Helsinki and the Institutional Review Board of Sichuan University West China Hospital. Written informed consent was obtained from all patients when they began treatment for osteoinductive CaP ceramics.

Perioperatively, the surgical strategies were summed up in three steps. Firstly, denosumab was administrated to each patient at least four dosages [2]. Secondly, all patients underwent preoperative arterial embolization to decrease the intraoperative blood loss [3]. Thirdly, a posterior midline approach was used for all patients. Tumor boundary was judged by computed tomography (CT) imaging before denosumab treatment [25]. To preserve neurologic function as much as possible, all patients were treated by extensive curettage. For adequate exposure, a cortical window was made, and the bony septa were broken by burr drill. If the GCT invaded the sacral nerve roots, the tumor was treated by piecemeal resection. The remaining cavity was filled with osteoinductive CaP ceramics in a uniform manner without impacting.

Postoperatively, CT imaging was accessed for each patient in regular follow-up. The radiographic lucency was used to evaluate the recurrence of the original lesion [26]. The healing status and quality of healing were evaluated by modified Neer classification [27] (Table 1). The common complications also were evaluated.

**Table 1.** Modified Neer classification of radiologic healing status

Type	Classification	Description
I	Healed	Cyst filled with new bone, with or without small radiolucent area(s) <1 cm in size
II	Healed with defects	Radiolucent area(s) <50% of the diameter of the bone with
III	Persistent cyst	Radiolucent area >50% of the diameter of the bone and with a thin cortical rim; no increase of the size of the cyst
IV	Recurrent cyst	Cyst reappeared in a previously obliterated area or a radiolucent area has increased in size

## Results

### Materials characterization

**Figure 1.**
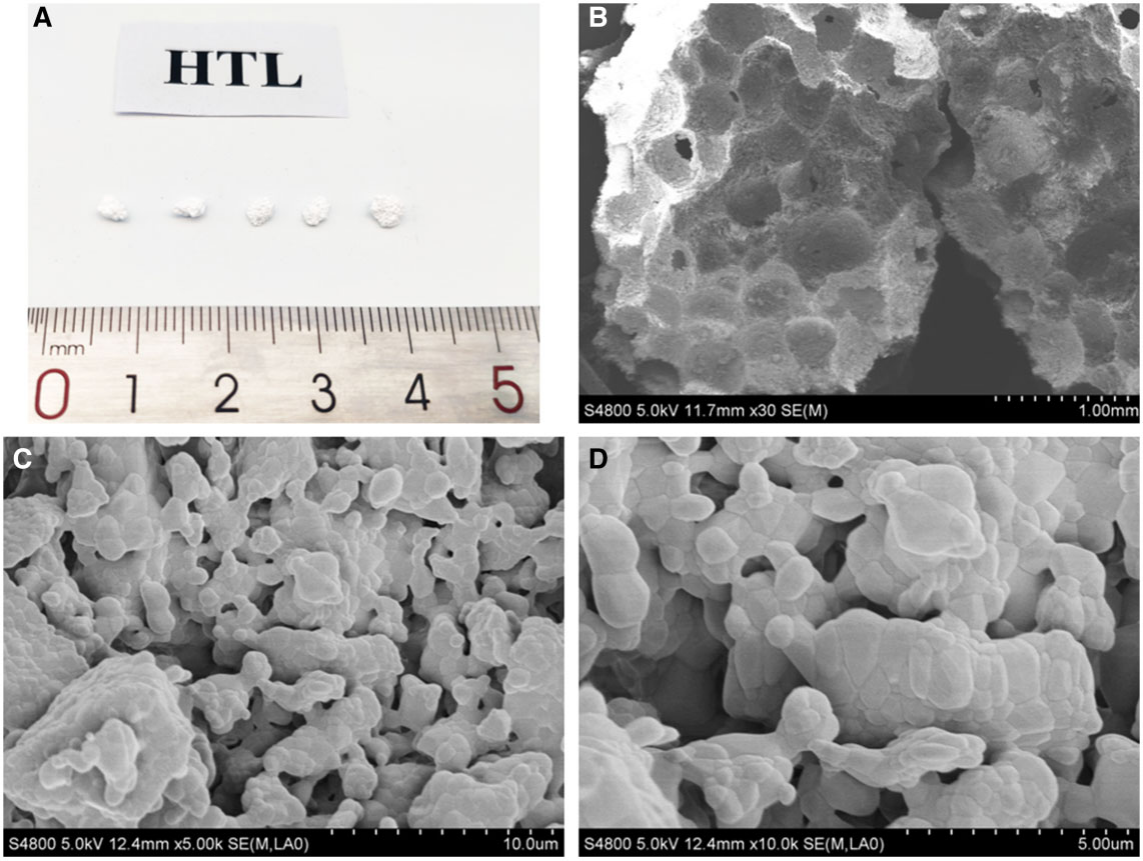
The macroscopic and microscopic morphologies of the osteoinductive CaP ceramics. The macroscopic morphology was showed in A, and the SEM showed the microscopic morphologies (B x30, C x5000, D x10000)

As demonstrated in Fig. 1, the granular CaP ceramics had irregular shape and the particle size was between 2.0 and 4.0 cm.

**Figure 2.**
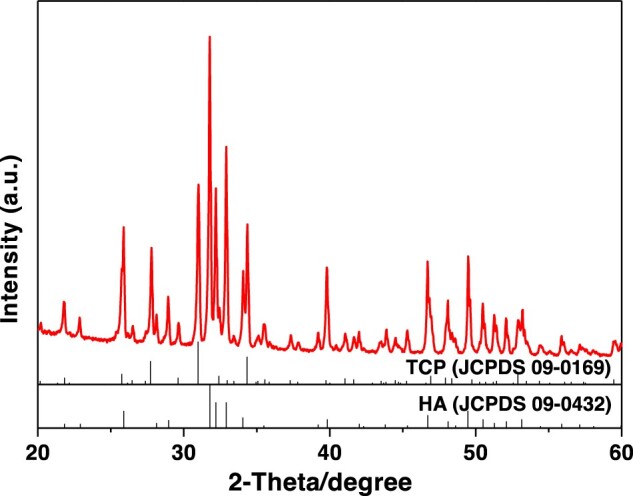
The XRD patterns

The ceramics had numerous interconnected macro-pores, on which abundant micro-pores were detected. Figure 2 shows the XRD pattern of the ceramic. It was confirmed that the ceramic was composed of HA and β-TCP phases.

By calculating, the HA phase ratio was 58% in the ceramic, while the β-TCP phase ratio was 42%. The porosity of the ceramic was 73.65% ± 1.24%.

### Animal experimental evaluation

Postoperatively, no incision healing problem was observed. Additionally, no superficial and deep wound infection was detected in all six beagles. Three months postoperative, all beagles were sacrificed with direct euthanasia. The CaP ceramics with the surrounding dorsal muscle was retrieved for histological evaluation.

**Figure 3.**
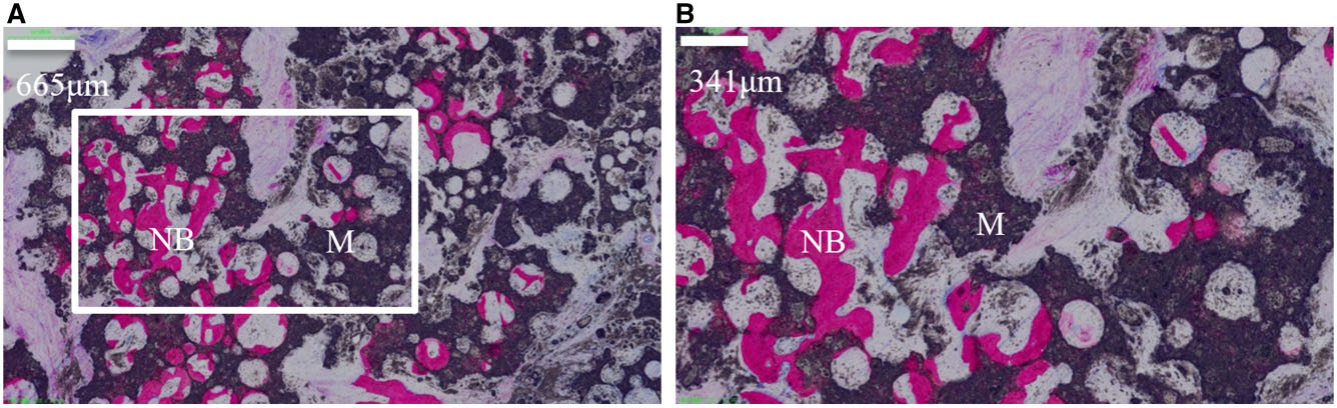
Histological analysis of bone formation after intramuscular implantation in beagles for 6 months [(**B**) is the partial enlarged drawing of (**A**)]. NB, new bone; M, residual material

New bone was detected in all samples with a 100% incidence rate of osteoinductivity. Despite the residual materials, the new bone was detected in defect areas and interconnected macro-pores with an irregular array. The quantitative analysis revealed that the average new bone area was 2.11% ± 1.23%, indicating an excellent osteoinductivity of the CaP ceramics. Figure 3 shows the new bone formation after intramuscular implantation with H&E staining.

### Clinical evaluation

**Figure 4.**
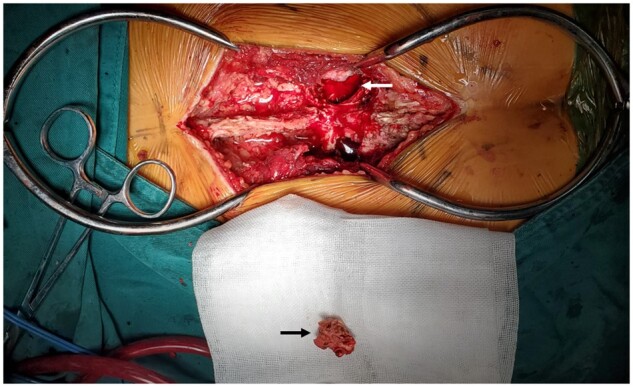
The surgical exposure for nerve-sparing surgery in sacrum. Left arrow, the tumor cavity in sarcum; right arrow, a cortical window

The operations on all six cases of sacral GCT patients were carried out successfully (Fig. 4).

After follow-up for 12 months, five cases obtained Type I, and one case had Type II healing status according to the modified Neer criterion [27] (Table 1).

**Figure 5.**
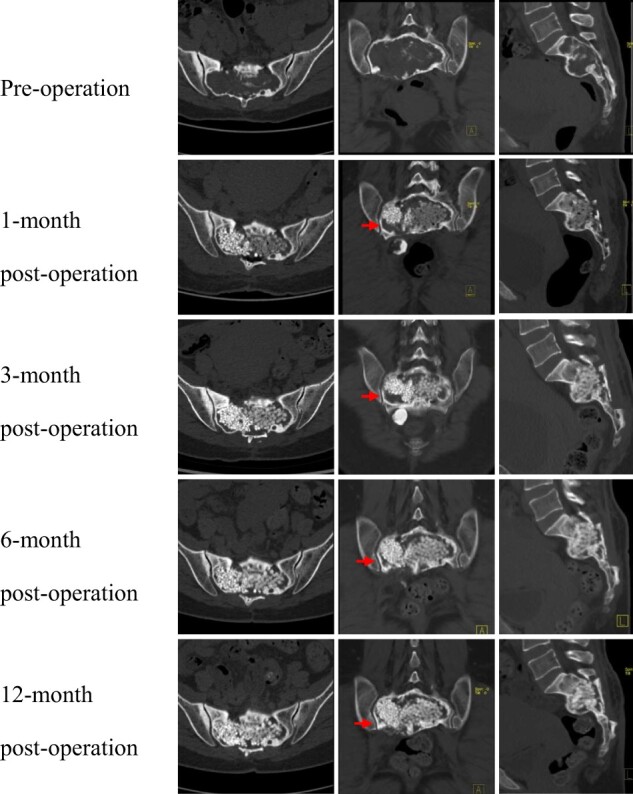
The CT scan of sacrum before and after surgery in Case 1. Arrow, new bone gradually filled the cavity

**Figure 6.**
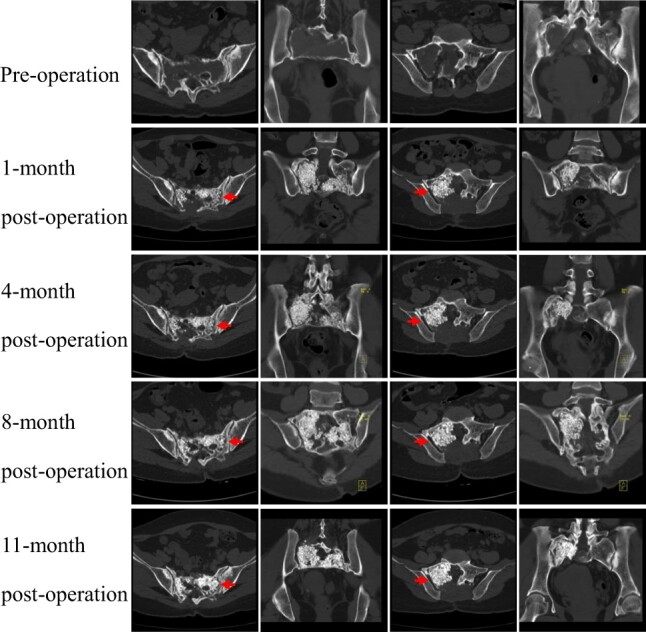
The CT scan of sacrum before and after surgery in Case 2. Left arrow, the CT attenuation value of the artificial bone was gradually increased; right arrow, the cortical rim was rebuilt

The median time of bone healing was 11.5 months. Healing initiation was detected in a median time of 3 months postoperatively, and the median time for relatively complete healing was 12 months. The ceramics were remodeled again according to the size and shape of the cavity and the thickness of the walls. The surrounding septa were closely reconnected together without pathological fracture. The absence of cortical rim, caused by GCT, was reconstructed by the ceramics and new bone. Figures 5 and 6 show the results of the CT scan preoperatively and postoperatively.

Postoperatively, 6-month CT detected that the ceramics and the boney walls were tightly bonded. Then, the density of the artificial bone was gradually increased in CT attenuation value (Fig. 5). Additionally, the bony bridge was observed among CaP ceramic granules with blurred boundaries. Moreover, the cortical rim was rebuilt in the right auricular surface of the sacrum. The cavity was gradually filled with new bone in the 12-month CT postoperatively.

At the latest follow-up, no infection was detected in all six patients. No radiographic lucency was observed on CT imaging, which indicated the local recurrence. The nerve function was completely preserved in all six patients without mild to severe limping.

## Discussion

As a new generation of bone substitute, CaP ceramics had similar chemical composition to bone minerals and exhibited excellent osteoconductivity and osteoinductivity [20]. CaP ceramics combined with autogenous bone marrow have been used in the management of bone defects caused by the appendicular benign tumor [28]. At a median of 36.4-month follow-up, 70.6% (24/34) patients had Neer I status, and 26.5% patients had Neer II status in radiological evaluation without related complications [28]. In 2018, Wu *et al.* [29] reported the completed healing rate of the graft was significantly improved in the β-TCP group (64.3%) according to modified Neer classification when compared to the allograft group (57.1%). A list of published core studies is summarized in Table 2.

**Table 2.** Summary of the most important published studies on ceramics in bone lesions following curettage

Author (year)	Disease	Patients (*n*)	Composition	Location	Follow-up (months)	Time of bone healing (months)	Quality of bone healing	Complications
El-Adl *et al.* (2009)^28^	Benign bone lesion	34	62.9% HA+33.9% TCP+3.2% gentamycin sulfate soaked with autogenous bone marrow	Extremities	36.4	5.0	70.6%—Neer I	Unrelated to the composite graft
							26.5%—Neer II	
							2.9%—Neer III	
Kokavec *et al.* (2010)^30^	Simple bone cyst	3	Pure β-TCP	Extremities	50.4	NM	100% complete healing	NM
Yang *et al.* (2014)^12^	Benign bone tumor	50	Calcium sulfate	Extremities	19.9	9.6	100% fusion and absorption	Swollen wound and delayed healing occurred (2)
								transiently temperature >38.5°C (4)
Wu *et al.* (2018)^29^	Benign bone tumor	44	Porous β-TCP	Extremities	19.6	4.9	60.7%—Neer I	Postoperative fracture (1)
							29.8%—Neer II	
							7.1%—Neer III	
Dragosloveanu *et al.* (2020)^11^	Benign bone lesions	8	65%HA+35% TCP	Extremities	12	5.1	62.5%—Neer I	No complications
							37.5%—Neer II	

HA, hydroxylapatite; TCP, tricalcium phosphate; NM, not mentioned.

The clinical healing results were significantly associated with the volume of remaining cavity, composition, weight-bearing and soft-tissue coverage significantly [11, 12, 29][30]. For sacral GCT, Khodamorad *et al.* [31] reported the use of cement or allograft in filling with the tumor cavity following nerve-sparing surgery. Unfortunately, no healing data were observed in this study. To our best knowledge, there were no prior reports on the effects of CaP ceramics on sacral GCT.

The clinical healing evaluation was categorized into four levels according to radiographic and clinical outcomes [12], including completed healing, healing, initial healing and nonunion. The clinical evaluation had three factors, including activities and weight-bearing and pain. For the radiographic assessment, modified Neer [27] and Capanna [32] classification were the most wildly used criteria. In this study, five patients had completed healing and one healed with defects. When compared to previous studies, our cases had a longer time of healing (median 11.5-month) than the results of those. We speculate that the longer time of healing might be attributed to the following reasons. Firstly, all the remaining cavities in the sacrum had a larger volume than those in the extremities. The volume of tumor cavity was closely related to healing status and time. In extremity, patients with tumor length <6.2 cm were 4.84 times more likely to achieve healing complete compared to those who had tumor length more than 6.2 cm [29]. Secondly, high-speed burring was used to obtain a safety margin and avoid local recurrence. Therefore, the cortical rim was inactivated by thermal damage, which took more time for osteogenesis. Thirdly, the nerve roots divided the remaining cavity into irregular areas, which became hard to completely fill with CaP ceramics. Most studies reported that the cystic lesions with CaP ceramics grafting healed following a centripetal pattern, in which the peripherally placed composite graft material was resorbed or replaced first and the more centrally placed material was the last to disappear [28, 33]. A similar pattern was observed in our cases. Peripheral CaP ceramics was resorbed firstly, then the gap of the cortical boundary was replaced by new bone (Figs 5 and 6).

Osteoinductivity of CaP ceramics can be optimized by its phase composition [20], which is one of the most important factors in inducing bone formation [21]. Recently, HA and β-TCP have been extensively studied for their osteoinductivity [20]. On the one hand, HA is the most stable biomaterial, with a low dissolution rate and excellent mechanical support. On the other hand, β-TCP with higher solubility is beneficial to the *in vivo* degradation of the ceramic. The previous study had demonstrated that biphasic CaP ceramics consisting of HA and β-TCP had better osteoinductivity than single-phase ceramics in intramuscular pockets of animal test [34, 35]. When compared to autogenous bone graft, osteoinductive CaP ceramics are equally efficient for bone repair in sheep model [36].

The pore structures including macro- and micro-pores play a critical role in the osteoinductivity of CaP ceramics. Given an optimal size, the diameter of macro-pore is within the range of 200–500 μm (Fig. 1). Among each macro-pore, the size of pore-interconnected channel is in the range of 100–200 μm [20]. Additionally, the diameter of micro-pores <10 μm plays a crucial role in promoting mass transport, cell adhesion and bone ingrowth [37]. The micro-pores on the walls of macro-pores promote the penetration of bodily fluids into the remaining cavity and producing rough surfaces on the walls, for cell attachment and the expression of osteogenic phenotype [20, 21]. Therefore, the intact structure of CaP ceramics is essential for its osteoinductivity. In our study, CaP ceramics was filled with the remaining cavity without impacting, which prevented the structural impairment. Meanwhile, the healing quality and process of revascularization was not correspondingly increased by conventional impaction technique [29]. However, we recommend no impaction for CaP ceramics in sacral GCT, which has nerve roots and bony septa.

For complications, radiographic lucency, which might be related to recurrence of GCT and foreign body reaction [26], was not detected in all six cases with short-term follow-up. Although conventional CaP ceramics may cause adverse soft-tissue reactions [38], no wound healing problems, inflammation and necrosis were found in this study. The osteoinductive CaP ceramics had similar inorganic components with human bone, which was related to the optimized biocompatibility and low complication rate [39]. Notably, despite the relatively short follow-up, no local recurrence was observed in our cases. We speculate that the degraded nano-scale HA (n-HA) products from osteoinductive CaP ceramics could suppress recurrence as followed. Firstly, n-HA has shown the capability of inhibiting tumor cells proliferation and sparing the normal cells by apoptosis pathway in various cancer [40], especially bone cancer [41]. Secondly, n-HA/scaffold was found to repair segmental bone defect under tumor environment [42]. Thirdly, n-HA/scaffold significantly inhibited the tumor growth and effectively prevented metastasis to the lung *in vivo*. The underlying mechanism was associated with multiple aspects of the tumor microenvironment, such as activating mitochondrial apoptosis pathway [42]. Further study is needed to illustrate this phenomenon *in vitro* and *invivo*.

## Conclusions

This study confirmed that the osteoinductive CaP ceramics had outstanding osteointegration and bone regeneration ability when used for filling the tumor cavity in sacral GCT. Following nerve-sparing surgery, the ceramic could be an excellent choice to manage large bone defects because of its osteoinductivity and osteoconductivity. In addition, it is convenient to use and can lead to considerable bone healing without severe complications in the sacral GCT. To obtain a more reliable clinical evaluation, further study with large cases and long-term follow-up are necessary.

## Ethics approval and consent to participate

This study was approved and monitored by the Ethical Committee of West China Hospital, Sichuan University in China (No.2019117). All patients signed the informed consent.
